# Physical activity and psychological support can replace “another pill” to manage cancer-related symptoms in children and adolescents diagnosed with cancer

**DOI:** 10.1186/s12906-024-04446-w

**Published:** 2024-04-22

**Authors:** Maxime Caru, Ariane Levesque, Smita Dandekar, Kathryn H. Schmitz

**Affiliations:** 1 Department of Pediatrics, Division of Hematology and Oncology, Penn State College of Medicine, 500 University Drive, Hershey, PA USA; 2 Department of Public Health Sciences, Penn State College of Medicine, Hershey, PA USA; 3 Department of Psychology, University of Montreal, Montreal, QC Canada; 4 Division of Hematology and Oncology, University of Pittsburgh, Pittsburgh, PA USA

## Abstract

The management of cancer-related symptoms with nonpharmacological treatment has been proven effective, but more studies are still required to strengthen the scientific evidence. Given the state of the evidence, one might wonder about the perceptions of pediatric oncology experts, healthcare providers and CAM providers regarding the use of supportive care in pediatric oncology. Related to this important question, Mora et al. recently published an exploratory qualitative study entitled “Supportive care for cancer-related symptoms in pediatric oncology: a qualitative study among healthcare providers” in the BMC Complementary Medicine and Therapies Journal. The data generated by the authors provided new insights and perspectives to the current literature. However, their findings must be put into perspective to increase the scope of the original article and to highlight that physical activity and psychosocial interventions are powerful nonpharmacological interventions to manage cancer-related symptoms.

We read with interest the exploratory qualitative study published in BMC Complementary Medicine and Therapies Journal by Dana C. Mora and colleagues [1] and wish to comment on some matters in this manuscript to provide additional insightful information about supportive care in pediatric oncology.

Children and adolescents diagnosed with cancer who have been treated with conventional medicines (chemotherapy, radiotherapy, surgery) have a 5-year overall survival rate greater than 85%. However, they often survive at the expense of several cancer therapy related adverse events affecting their health, and long-term morbidity and mortality. Late effects include a spectrum of health-related complications including fatigue, nausea, pain, depression symptoms, anxiety, sleep disturbance, and physical limitations [2, 3]. The majority of children and adolescents diagnosed with cancer will have at least 1 late effect, which can be severe or even life threatening in about 25% of childhood cancer survivors [4]. Thus, the growing number of diagnoses and survivors, along with a better understanding of the long-term effects of cancer therapies, has led to new complementary and alternative medicine (CAM) modalities, such as physical activity, nutrition and psychosocial support. Although the management of cancer-related symptoms with nonpharmacological treatment has been proven effective, more studies are still required to strengthen the scientific evidence. Given the state of the evidence, one might wonder about the perceptions of pediatric oncology experts, healthcare providers and CAM providers regarding the use of supportive care in pediatric oncology.

Related to this important question, Mora et al., recently published an exploratory qualitative study [1]. The authors conducted 22 semi-structured individual interviews to better understand the philosophical and medical context of supportive care modalities, including complementary and alternative medicine modalities in children and adolescents diagnosed with cancer. Participants interviewed were pediatric oncologists (*N* = 6), nurses (*N* = 5), acupuncturists (*N* = 5), healers (*N* = 3), nutritionists (*N* = 2), physiotherapist (*N* = 1), play therapist (*N* = 1), massage therapist (*N* = 1), music therapist (*N* = 1), psychodrama therapist (*N* = 1), anthroposophic medicine (*N* = 1) and homeopath (*N* = 1) with clinical experience in pediatric supportive care from Canada, the Netherlands, Norway, Germany, and the United States. The authors recognized that all the participants interviewed were from high-income countries which could limit the generalizability of the results to low, lower-middle and upper-middle countries.

The data generated by the authors provided new insights and perspectives to the current literature [1]. However, their findings must be put into perspective since they might give a biased overview of the perceptions of experts and providers regarding the use of supportive care in pediatric oncology since some of the most empirically supported disciplines by the scientific literature in pediatric oncology were not included. Indeed, even though physical activity and psychological support emerge as core CAM interventions, no experts in these fields were included in the study by Mora et al. [1, 5, 6]. The lack of inclusion of these important disciplines therefore restricted their qualitative analysis which could have benefited from the inclusion of these perspectives to give a complete overview of supportive care in pediatric oncology. It was also surprising to read that two oncologists interviewed were skeptical about the use of CAM modalities due to the lack of evidence. This response is understandable considering that it appears that the authors did not thoroughly define what they meant by “CAM modalities” within the supportive care system in pediatric oncology. Hence, participants were only able to provide their general opinion on the matter, despite the fact that there is a variety of CAM modalities that have been explored in pediatric oncology [7].

A powerful statement from one of the oncologists was as follows: “There are symptoms like fatigue, anxiety, insomnia, that we just don’t have the interventions for. I am thinking there has got to be a better way to make people feel better as they’re going through their cancer treatments that doesn’t just involve asking, particularly children, to take more medicines” [1]. Fortunately for children and adolescents diagnosed with cancer, there are other interventions for them to feel better that do not require taking another pill. Indeed, evidence-based medicine has shown important improvements in the management of a myriad of cancer-related symptoms with physical activity, nutrition and psychosocial support in children and adolescents diagnosed with cancer [8–10]. The recent International Pediatric Oncology Exercise Guidelines highlighted that physical activity should be integrated within the standard care of pediatric oncology due to its several benefits on children and adolescents’ health to manage and improve their long-term symptoms [11]. These guidelines also align with the American College of Sports Medicine recommendations for cancer survivors highlighting that exercise is medicine in oncology [12]. It has also been argued that psychological support should emerge as standard care in pediatric oncology due to its effectiveness in reducing emotional distress in both children and adolescents, and their parents [13]. It is therefore disappointing to note that no exercise physiologists or psychologists were interviewed in Mora’s study and that the authors did not discuss physical activity or psychological support as a CAM modality in their paper, despite their high level of evidence in pediatric oncology.

We could not agree more with the authors, however, that it is important to provide accurate information regarding CAM modalities that come from reliable sources, such as pediatric oncology experts. Since physical activity and psychological support emerge as some of the CAM interventions with the most empirical support [5, 6], experts in these fields need to be consulted for these supportive interventions to be systematically implemented in a rigorous manner. Indeed, despite strong evidence supporting their benefits, patients are not yet systematically referred to exercise physiologists and psychologists. To illustrate, only 1 out of 5 oncology experts refer their patients to physical activity programs [14], even though almost 4 out of 5 report being willing to recommend to their patients to be more active [15]. Nevertheless, oncology experts are often aware of the potential benefits of physical activity. Indeed, a study found that almost 90% of physicians recommend physical activity to their patients, while the other 10% recognize their lack of training in this area, which limits their expertise to recommend physical activity to their patients [16]. Regarding psychological support, in the early 2000s, one study found that only 32% of pediatric cancer patients were referred for psychological consultation [17]. Although this rate is likely higher today, referring families to psychologists with experience in pediatric oncology remains of utmost importance, especially considering the unique psychological issues that can be faced by children and adolescents diagnosed with cancer.

In conclusion, the study of Mora et al. provides insightful information on clinical care, clinical care guidelines, and policy-making decisions for cancer-related symptoms on the sole condition that all CAM modalities are taken into account. Doing so will contribute to improving supportive care in pediatric oncology and educate the key players in this field. It will also contribute to giving parents and patients valuable information to improve their general wellbeing and gain control over their cancer-related symptoms. Physical activity and psychosocial interventions are powerful nonpharmacological interventions to manage cancer-related symptoms that should be routinely offered to children and adolescents diagnosed with cancer.

## Data Availability

Data sharing not applicable to this article as no datasets were generated or analysed during the current study.
